# Stomatal development in the cycad family Zamiaceae

**DOI:** 10.1093/aob/mcab095

**Published:** 2021-07-15

**Authors:** Mario Coiro, Maria Rosaria Barone Lumaga, Paula J Rudall

**Affiliations:** 1 Department of Systematic and Evolutionary Botany, University of Zurich, Zurich, Switzerland; 2 Ronin Institute for Independent Scholarship, Montclair, NJ, USA; 3 Department of Biology, University of Naples ‘Federico II’, Naples, Italy; 4 Royal Botanic Gardens, Kew, Richmond, UK

**Keywords:** Zamiaceae, stomata, ecophysiology, development, *Stangeria*, *Bowenia*, *Zamia*, *Dioon*, *Macrozamia*, *Ceratozamia*

## Abstract

**Background and Aims:**

The gymnosperm order Cycadales is pivotal to our understanding of seed-plant phylogeny because of its phylogenetic placement close to the root node of extant spermatophytes and its combination of both derived and plesiomorphic character states. Although widely considered a ‘living fossil’ group, extant cycads display a high degree of morphological and anatomical variation. We investigate stomatal development in Zamiaceae to evaluate variation within the order and homologies between cycads and other seed plants.

**Methods:**

Leaflets of seven species across five genera representing all major clades of Zamiaceae were examined at various stages of development using light microscopy and confocal microscopy.

**Key Results:**

All genera examined have lateral subsidiary cells of perigenous origin that differ from other pavement cells in mature leaflets and could have a role in stomatal physiology. Early epidermal patterning in a ‘quartet’ arrangement occurs in *Ceratozamia*, *Zamia* and *Stangeria*. Distal encircling cells, which are sclerified at maturity, are present in all genera except *Bowenia*, which shows relatively rapid elongation and differentiation of the pavement cells during leaflet development.

**Conclusions:**

Stomatal structure and development in Zamiaceae highlights some traits that are plesiomorphic in seed plants, including the presence of perigenous encircling subsidiary cells, and reveals a clear difference between the developmental trajectories of cycads and Bennettitales. Our study also shows an unexpected degree of variation among subclades in the family, potentially linked to differences in leaflet development and suggesting convergent evolution in cycads.

## INTRODUCTION

**Fig. 1.**
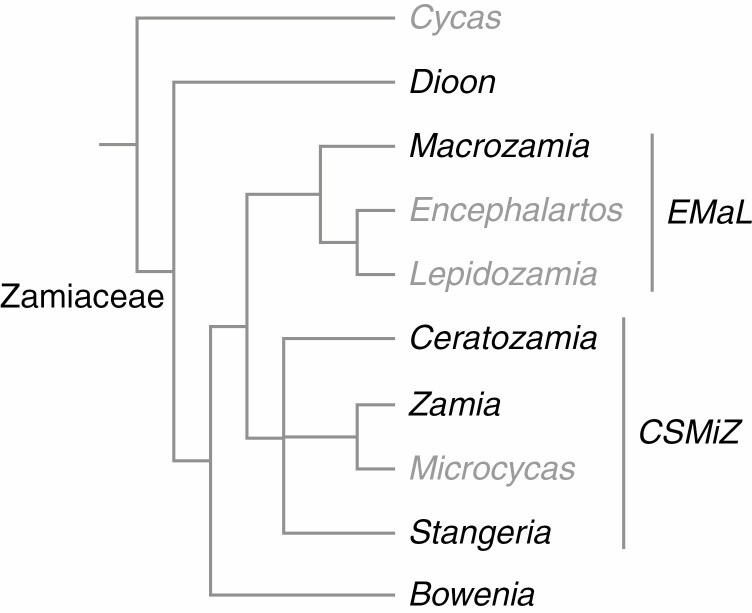
Phylogeny of the cycads based on molecular results cited in the text. Genera sampled for our developmental analyses are in black.

The extant gymnosperms, although relatively species-poor compared with angiosperms, display a high degree of morphological disparity that reflects a long evolutionary history, extending as far back as the Devonian period of the Palaeozoic era. Extensive extinctions among all gymnosperm groups (Crisp and Cook, 2011) have removed putative morphological intermediates, making the fossil record potentially important for our understanding of the evolution of seed plants. Among the four gymnosperm lineages that include living representatives, the cycad lineage is highly significant in studies of character evolution because of its phylogenetic placement close to the root node of the spermatophyte clade, either as sister to all other extant gymnosperms in most molecular analyses (e.g. Graham and Iles, 2009; Ran *et al.*, 2018) or as sister to all other extant seed plants in morphological analyses (e.g. Doyle, 2006; Hilton and Bateman, 2006). Cycads display many traits that are considered plesiomorphic among seed plants (Brenner *et al.*, 2003) and are thus pivotal in helping to resolve relationships among fossil and extant groups. Among the extant cycads, in molecular phylogenies (e.g. Salas-Leiva *et al.*, 2013) the genus *Cycas* L. (Cycadaceae) is placed as sister to the other nine extant genera (family Zamiaceae). *Cycas* is also relatively divergent morphologically from the other cycads (Table 1); for example, all cycads possess compound leaves, but *Cycas* leaflets display circinate vernation and a single central vein, compared with erect ptyxis and multiple veins in Zamiaceae (Stevenson, 1981). Relationships among the genera of Zamiaceae are incompletely resolved; in some analyses *Dioon* is sister to the rest and *Bowenia* is sister to two further clades, here informally termed the *CSMiZ* clade and the *EMaL* clade (Fig. 1).

**Table 1.** Details of material examined

Species	Stage	Collection numbers	Site grown
*Bowenia spectabilis* Hook.	Seedling grown from seeds from commercial source (www.rarepalmseeds.com)	XX-0-Z-20190803	University of Zurich, Zurich, Switzerland
*Bowenia spectabilis*	Adult leaflets	9367*A	Montgomery Botanical Center, Coral Gables, FL, USA
*Ceratozamia hildae* G.P.Landry & M.C.Wilson	Developing leaflets from adult specimens	1978-1827	Royal Botanic Gardens, Kew, UK
*Cycas thouarsii* R.Br. ex Gaudich.	Adult specimens	417-1-1	Orto Botanico di Napoli, Naples, Italy
*Cycas circinalis* L.	Adult specimens	414-0-1	Orto Botanico di Napoli
*Dioon edule* Lindl.	Developing and adult leaflets from adult specimens	CAL31	Orto Botanico di Napoli
*Macrozamia communis* L.A.S.Johnson	Seedling grown from seeds from commercial source (www.rarepalmseeds.com)	XX-0-Z-20190808	University of Zurich
*Stangeria eriopus* (Kunze) Baill.	Developing leaflets from adult specimens	651325*F, 2000530*A, 651325*F, 80727*G	Montgomery Botanical Center
*Zamia integrifolia* L.f.,	Developing leaflets from adult specimens	XX-0-Z-20190811	University of Zurich
*Zamia roezlii* Regel	Developing leaflets from adult specimens	XX-0-Z-20190812	University of Zurich

Stomatal morphology has potential to help resolve the relationships among these genera and also between extant and fossil gymnosperms. Cuticles and stomata are sometimes well preserved in compression fossils, and the phylogenetic signal of stomatal traits has long been recognized (e.g. Porsch, 1905; Florin, 1931, 1933). However, descriptions based entirely on mature stomata are potentially non-homologous; for example, paracytic stomata (which possess a pair of lateral subsidiary cells) can have different developmental trajectories in different taxa. Thus, developmental studies are important to clarify homologies. One of the most influential distinctions was made by Florin, who characterized ‘haplocheilic’ and ‘syndetocheilic’ stomatal complexes in both living and fossil seed plants (Florin, 1931). This differentiation was explicitly based on development (see also Rudall and Bateman, 2019). In haplocheilic stomata, a protodermal cell functions directly as a guard-mother cell (GMC) with no prior divisions; modified subsidiary cells (where present) are derived from surrounding cells, which are termed perigene cells. In syndetocheilic stomata, a protodermal cell becomes a meristemoid that divides asymmetrically to form a GMC and one or more specialized neighbour cells, which are termed mesogene cells.

**Fig. 2.**
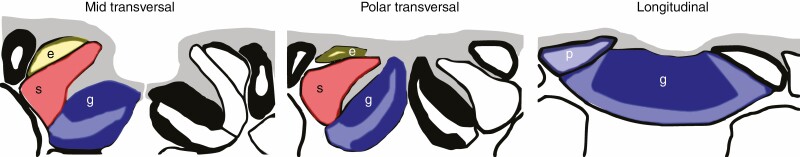
Schematic generalized drawings of stomata of Zamiaceae in middle transverse, polar transverse and longitudinal section. Cell wall in black, cuticle in grey. The guard cells (g, in dark blue), subsidiary cells (s, in red), encircling cells (e, in yellow) and polar cells (p, in light blue) are highlighted.

Mature stomata in cycads are characterized by a ring of cells surrounding the guard cells (Fig. 2), typically arranged in a single layer at the poles and two to four cell layers at the lateral sides (Florin, 1931, 1933; Greguss, 1957, 1968; Bobrov, 1962; Pant and Nautiyal, 1963; Griffith *et al.*, 2014; Magellan *et al.*, 2018; Vovides *et al.*, 2018; Coiro *et al.*, 2020). In many cycad species, the stomata are sunken in deep stomatal pits (Florin, 1931; Greguss, 1957; Pant and Mehra, 1964). This three-dimensional structure makes studies of development highly problematic in some cycads because they cannot readily be imaged in surface view; few photomicrographs exist of stomatal development.

In this paper, we evaluate traditional hypotheses on the origin of the subsidiary cells. We use a range of anatomical techniques to describe the development of the leaflet epidermis in six cycad species representing five genera, including all major clades of Zamiaceae. Previous detailed studies of stomatal development are restricted to *Cycas* and *Dioon* Lindl. (Florin, 1931; Pant and Mehra, 1964), and variation within Zamiaceae is hitherto unknown.

## MATERIALS AND METHODS

### Terminology of cycad stomata

In his description of the stomata of *Dioon edule*, Florin (1931) identified two different cell types in the stomatal apparatus: Nebenzellen (neighbour cells, directly flanking the guard cells) and Kranzzellen (crown cells, at the poles of the guard cells, as well as the three layers of cells overlying the Nebenzellen). Harris (1932) translated Florin’s terminology into English, interpreting the Nebenzellen and the polar Kranzellen as subsidiary cells and the lateral Kranzzellen as encircling cells. Greguss (1968) instead translated Nebenzellen as neighbour cells and Kranzzellen as accessory cells. However, Harris’ terminology has been more widely used in subsequent studies of cycad stomata (Pant and Nautiyal, 1963; Pant and Mehra, 1964; Barone Lumaga *et al.*, 2015; Vovides *et al.*, 2018). To avoid confusion about the use of subsidiary cells, we follow the nomenclature from Coiro *et al.* (2020), which distinguished between subsidiary cells and polar cells (Fig. 2).

### Material examined

The material examined is listed in Table 1.

### Methods

**Fig. 3.**
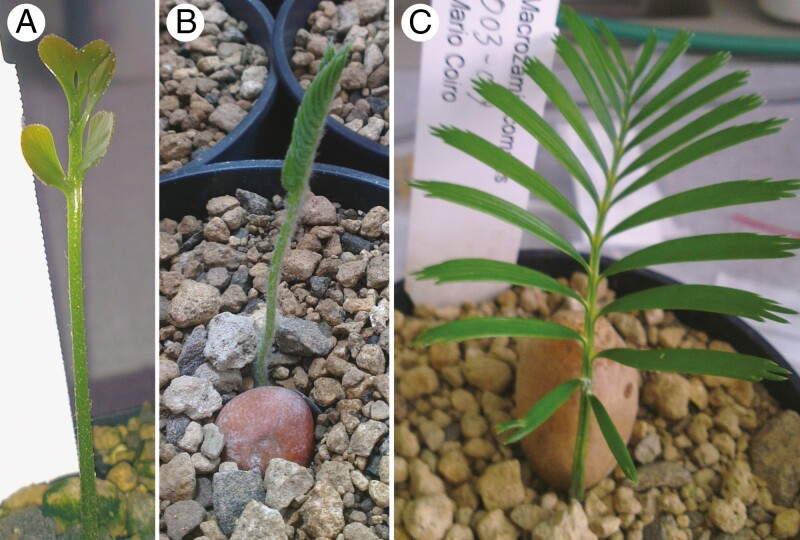
Photographs of germinated seedlings of (A) *Bowenia spectabilis*, (B) *Dioon edule* and (C) *Macrozamia communis*.

Seeds of four species (Table 1) were germinated on a perlite substrate and transferred to pumice after the emergence of the first prophyll before sampling the first developing leaflets (Fig. 3). Leaflet structure differs between species examined (Table 2; Fig. 3). In all species, leaflets were sampled at different developmental stages and immediately fixed in formalin–acetic acid–alcohol. They were subsequently transferred to 70 % ethanol and stored at 4 °C. Material for sectioning was embedded in Kulzer’s Technovit 7100 (2-hydroethyl methacrylate) as described by Igersheim and Cichocki (1996). This method involves dehydration of the samples in an ethanol series and stepwise infiltration with the following ratios of 100 % ethanol : Technovit solution: 50 : 50, 25 : 75, 0 : 100. The embedded specimens were sectioned on a Microm HM 355 rotary microtome using a conventional microtome knife D. Sections of mostly 2 µm were stained with ruthenium red and toluidine blue and mounted on microscope slides in Histomount. Non-embedded sections of leaflets were mounted in 80 % glycerol or Hoyer’s solution, prepared according to Coiro and Truernit (2017). Sections of mature leaflets of *D. edule* and *Bowenia spectabilis* were subject to pseudo-Schiff propidium iodide (PS-PI) staining, a technique that allows confocal imaging of cell walls, as described in Coiro and Truernit (2017), and then mounted in Hoyer’s solution.

**Table 2.** Leaflet structure in cycad genera examined

Species	Leaflet structure
*Bowenia*	Adult leaflets bipinnate with circinnate ptyxis, but first photosynthetic leaf (eophyll) simply pinnate with conduplicate ptyxis (i.e. leaflets face each other adaxially). The leaflets are spatulate early in their development (Fig. 3A).
*Ceratozamia*	Leaf ptyxis reflexed; leaflets of each side overlap each other, with the adaxial side of the proximal pinna facing the adaxial side of the distal pinna.
*Cycas*	Circinate vernation and a single central vein
*Dioon*	Leaf ptyxis erect; leaflets of each side overlap each other, with the adaxial side of the proximal pinna facing the adaxial side of the distal pinna (Fig. 3B).
*Macrozamia*	Leaves simply pinnate, with erect ptyxis of the whole leaves; leaflets of each side overlap each other, with the adaxial side of the proximal pinna facing the adaxial side of the distal pinna.
*Stangeria*	Leaves simply pinnate, each leaflet possessing a clear midrib. Leaf ptyxis strongly inflexed. The primordia of the leaflet midrib are conduplicate, and the lamina of each leaflet is also conduplicate.
*Zamia*	Leaves simply pinnate, with plicate leaflets in *Z. roezlii*. Leaf ptyxis reflexed; leaflets overlap each other.

For examination of mature structure, cuticles of mature leaflets of *Bowenia serrulata*, *Cycas circinalis* and *Cycas thouarsii* were isolated after maceration in a mixture of H_2_O_2_ and 80 % ethanol. Light and fluorescence micrographs were obtained using a Zeiss Axioscope using a brightfield filter. PS-PI-stained samples of *B. spectabilis* were observed using a Leica TCS SP8 microscope. Excitation was obtained using either 405-nm excitation and a DAPI emission filter or 488-nm excitation and a PI emission filter.

## RESULTS

### Bowenia

**Fig. 4.**
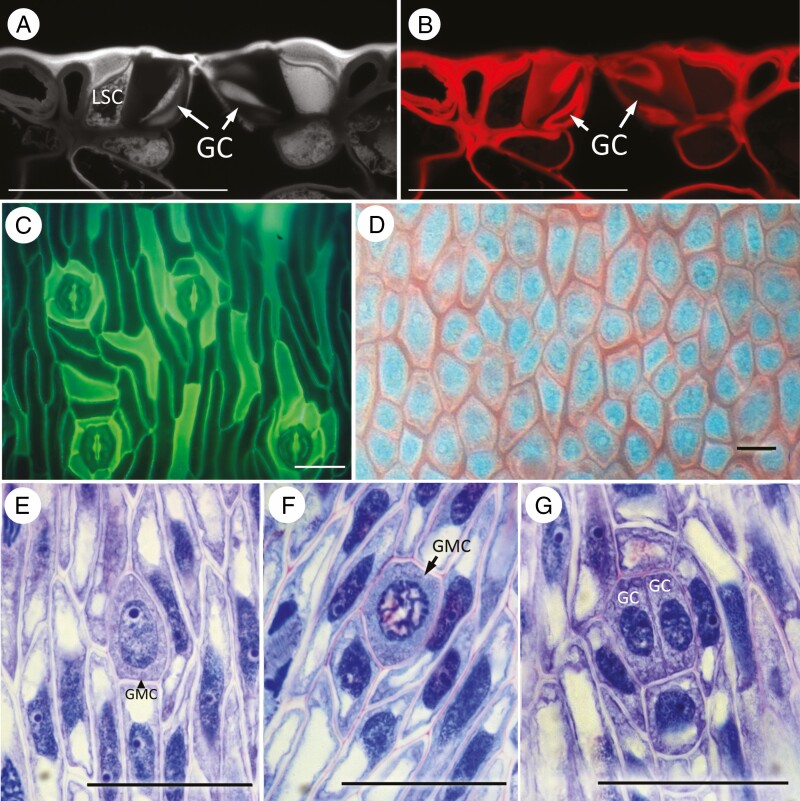
*Bowenia spectabilis.* (A, B). Transverse section of stomatal complex of an adult leaflet stained with pseudo-Schiff-propidium iodide observed using confocal laser scanning microscopy and imaged using (A) UV excitation, (B) propidium iodide excitation. (C) Fluorescence micrograph of isolated cuticle from adult leaflet stained with Auramine O, showing mature stomata in axial cell files. (D) Fluorescence micrograph of isodiametric protodermal cells in developing leaflet. (E–G) Development of stomatal complexes at different stages. Scale bars = 50 µm. Abbreviations: GMC, guard mother cell; GC, guard cell; LSC, lateral subsidiary cell.

In *B. spectabilis* (Fig. 4), mature stomata are all similarly orientated parallel to the leaflet axis and the guard cells are not sunken in a stomatal pit (i.e. they are flush with the surface: Fig. 4A, B). Each stoma has two to five subsidiary cells (Fig. 4C). Mature subsidiary cells have a thicker cuticle than surrounding pavement cells and contain denser cytoplasm. Mature guard cells contact the subsidiary cells on their dorsal wall and contact the specialized cells of the substomatal apparatus on their inner wall (Fig. 4A, B). Guard cells show differentiation of the cell wall (potentially lignin or pectin impregnation) on the dorsal and ventral sides of the cytoplasm (Fig. 4B).

Early in development, the protodermal cells are isodiametric (Fig. 4D); they commence axial elongation before division of the GMC into two guard cells. The GMCs differentiate directly from enlarged protodermal cells (Fig. 4E–G). Subsidiary precursor cells differentiate from the cell files adjacent to the GMCs, and sometimes undergo oblique divisions.

### Ceratozamia

**Fig. 5.**
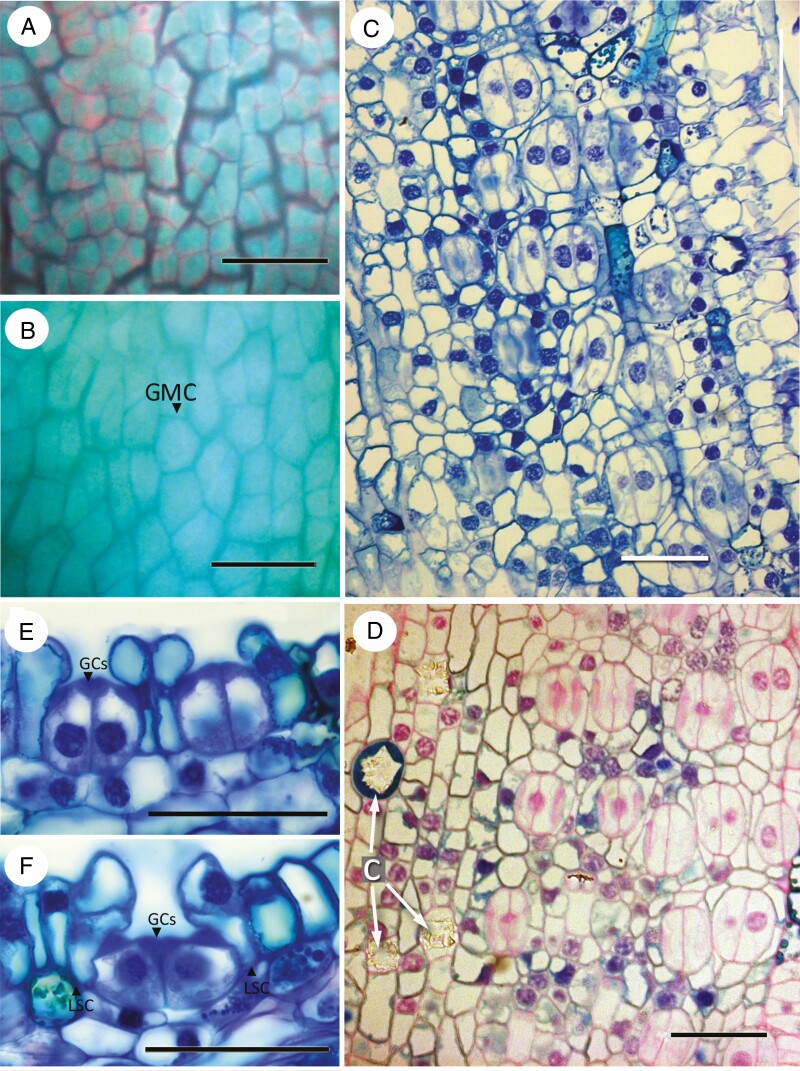
(A, B) *Ceratozamia hildae*; (C–F) *Dioon edule*. (A) Early development showing squared (quartet) arrangement of protodermal cells. (B) Slightly later stage, indicating protodermal cell enlarging to form a guard-mother cell (GMC). (C, D) Tangential sections of developing leaflets showing developing stomata in an intercostal stomatal band, all similarly orientated in axial cell files along the leaflet axis. Crystals are present in cells over veins in the slightly later stage in D. (E, F) Transverse sections of leaflets showing stomata; neighbour cells elongating periclinally in E and divided in F. Scale bars = 50 µm. Abbreviations: C, crystal; GMC, guard mother cell; GC, guard cell.

Early development was observed in *Ceratozamia hildae* (Fig. 5A, B). Protodermal cells undergo both longitudinal and perpendicular divisions, resulting in a squared (quartet) arrangement (Fig. 5A). The GMCs are isodiametric and originate by direct enlargement of one of the protodermal cells (Fig. 5B).

### Dioon

Later development was observed in *Dioon* (Fig. 5C–F). Stomata develop in irregular cell files in the intercostal regions, resulting in a closely spaced arrangement with the apertures orientated parallel to the leaflet axis (Fig. 5C, D). Subsidiary cells develop from axially elongated neighbour cells, which divide obliquely leading to distal encircling cells (Fig. 5E, F). In the mature stomatal apparatus, three encircling cells with thick cell walls are placed distally to the subsidiary cells, which have a thinner wall than other pavement cells. The subsidiary cells contact the dorsal outer wall of the guard cells and the guard cells are enclosed in a stomatal pit.

### Macrozamia

**Fig. 6.**
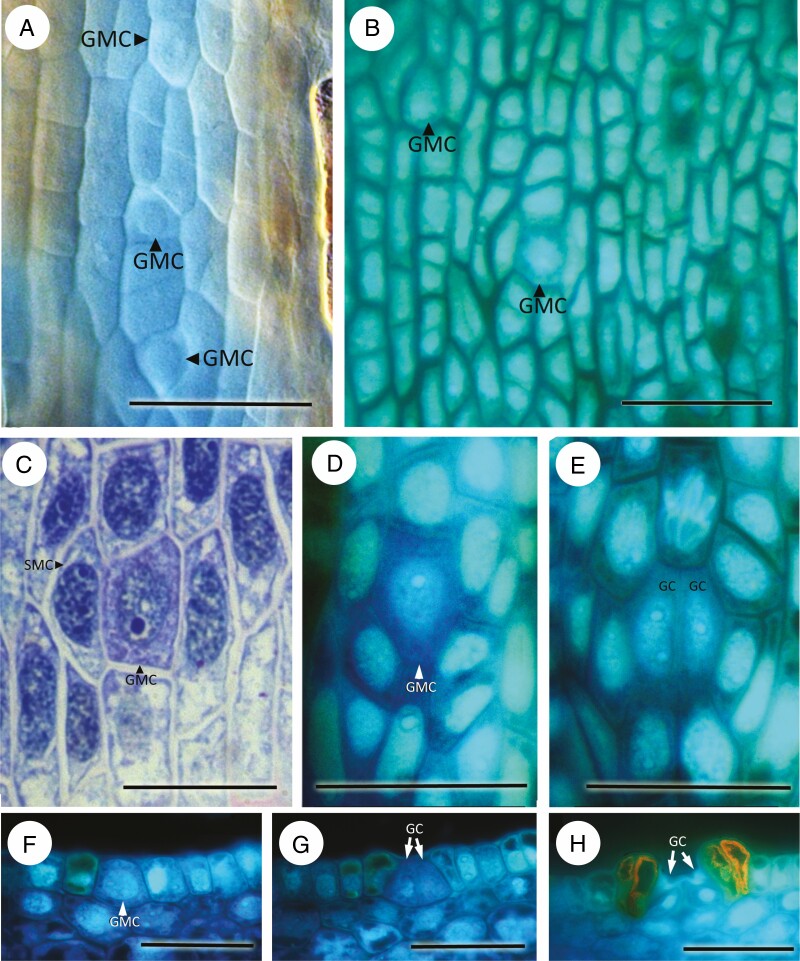
*Macrozamia communis*, developing leaflets imaged using (A) differential interference contrast, and (B, D–H) fluorescence micrography. (A, B) GMCs in axial cell files. (C–E) Stomatal development in surface view showing (C, D) GMCs and (E) guard cells. (F–H) Stomatal development in transverse section at successive stages. Scale bars = 50 µm. Abbreviations: GMC, guard mother cell; GC, guard cell.

In *Macrozamia communis* (Fig. 6), protodermal cells are isodiametric or slightly axially elongated, with some dichotomies and anastomoses leading to a more irregular arrangement (Fig. 6B); epidermal cells become more axially elongated during later development. GMCs are oval or square and share the same lineage as one of the polar cells, which is therefore mesogenous (Fig. 6A). Other subsidiary cells originate from neighbour cells (Fig. 6D–H). Two neighbour cells can divide further to give rise to three to five encircling cells; further divisions in the lateral neighbour cells result in two rings of proximal subsidiary and distal encircling cells. During development, the neighbour cells also elongate distally, resulting in a stomatal pit (Fig. 5H).

### Stangeria

**Fig. 7.**
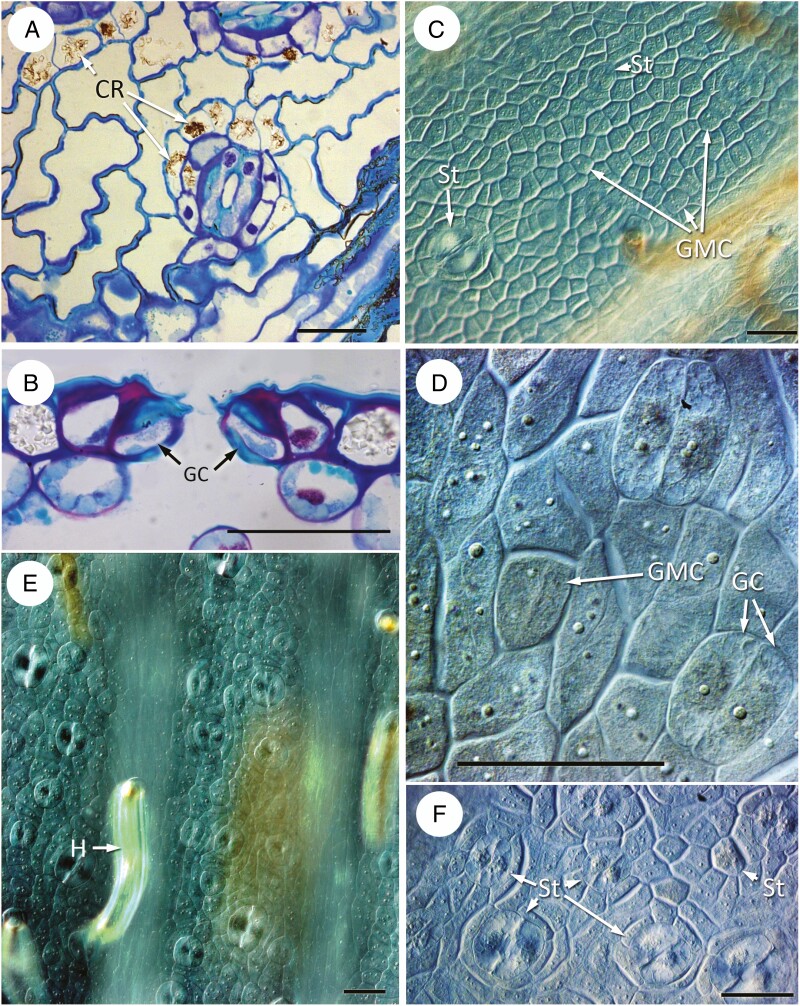
*Stangeria eriopus*. (A) Paradermal view of mature epidermis showing stoma with guard cells containing cytoplasm and nuclei; encircling subsidiary cells also with active nuclei. Calcium oxalate crystals present in surrounding intercostal epidermal cells. (B) Transverse section of stomatal complex showing guard cells with thickened cell walls containing lignin–pectin deposits, and encircling cells with calcium oxalate crystals. (C–F) Fluorescence and differential interference contrast images of cleared leaflets showing stomatal development. (C) Protodermal cells interspersed with both GMCs and stomata. (D) Slightly later stage, with guard cells and a GMC undergoing division. (E) Leaf clearing showing both differentiated and developing stomata in intercostal regions, most similarly axially orientated. Costal region (in different focal plane) with trichomes (hairs). (F) Later stage showing similarly orientated differentiated stomata; some smaller stomata in different orientation. Scale bars = 50 µm. Abbreviations: Cr, crystal; GMC, guard mother cell; GC, guard cell; St, stomata.

In *Stangeria eriopus* (Fig. 7), protodermal cells are arranged in irregular squares or rectangles, lacking a clear direction of division (Fig. 7C). GMC differentiation commences early in leaflet differentiation and continues throughout leaf development, resulting in stomatal complexes at different developmental stages in close proximity to each other (Fig. 7D–F). Division and differentiation of new GMCs continues until late in leaflet differentiation, resulting in intercostal axial rows of stomata. Early-formed stomata are mostly axially orientated, but later-developing stomata are often randomly orientated (Fig. 7D–F). Subsidiary cells are formed from neighbour cells. They undergo divisions parallel to the margin of the guard cells, resulting in subsidiary cells (Fig. 7A). Pavement epidermal cells often have slightly sinuous walls in surface view. Subsidiary cells differ from pavement cells in their shape; some also maintain a nucleus and cytoplasm and have a slightly thicker cuticle (Fig. 7A, B). Crystals are often present in the epidermal cells, often in the polar cell and encircling cells (Fig. 7A). Mature guard cells are flush with the surface; they have a thickened cell wall both dorsally and ventrally; and they contact the subsidiary cells and some mesophyll cells on their dorsal wall (Fig. 7B).

### Zamia

**Fig. 8.**
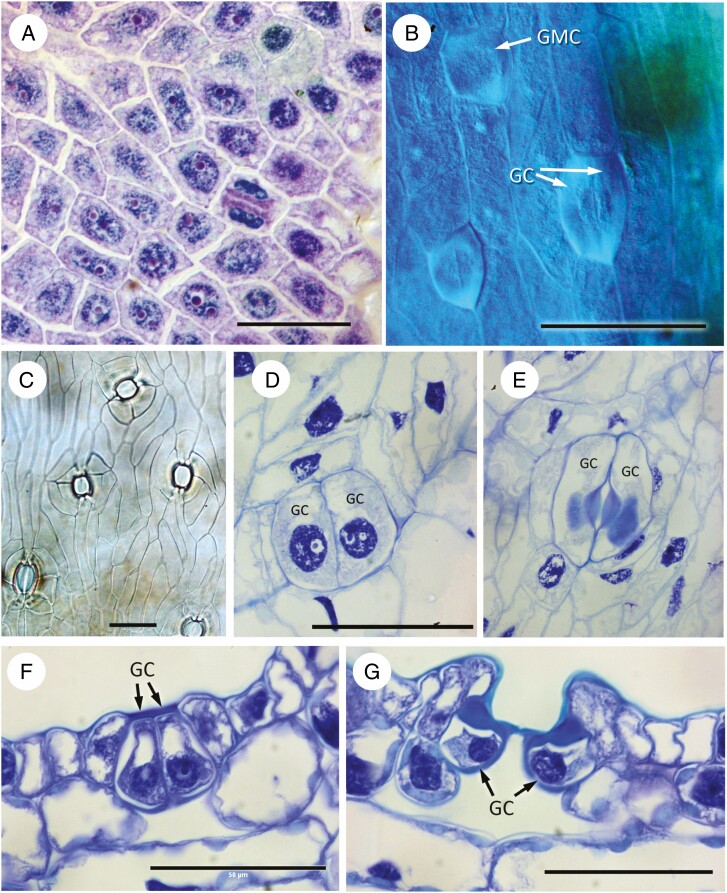
*Zamia*. (A, B) *Zamia integrifolia*, early developmental stages; (C–G) *Zamia roezlii*, differentiated stomata. (A) Protodermal cells. (B) Early stage with GMCs undergoing division, arranged in axially orientated cell files. (C) Surface view showing stomatal openings surrounded by encircling cells. (D) Young stoma with dividing lateral subsidiary cells. (E) Mature stoma with encircling cells and wall thickenings on guard cells. (F, G) Transverse sections showing successive stages of maturing stomata with guard cells already differentiated; in G the guard cells are sunken due to enlargement of the encircling subsidiary cells. Scale bars = 50 µm. Abbreviations: GMC, guard-cell mother cell; GC, guard cell.

In *Zamia* (Fig. 8), early development was observed in *Z. integrifolia* (Fig. 8A, B) and later development in *Z. roezlii* (Fig. 8C–G). Protodermal cells are angular and isodiametric (Fig. 8A), soon becoming axially elongated (Fig. 8B). GMCs are angular in surface view (Fig. 8B), although they can appear more oval later in development. Subsidiary cells develop from cells flanking the GMC. Later in development, stomata are orientated parallel to the leaflet axis (Fig. 8C–E). Neighbour cells undergo divisions, resulting in subsidiary cells. Subsidiary cells also elongate distally to form a stomatal pit (Fig. 7G).

## DISCUSSION

Our investigation of stomatal and epidermal development and stomatal anatomy in six of the nine genera of Zamiaceae has highlighted traits shared with other gymnosperm groups as well as potentially synapomorphic traits of the cycads such as the presence of encircling cells and sunken stomata. It has revealed a clear difference between the developmental trajectories of cycads and Bennettitales. The anatomy and consistent presence of subsidiary cells opens the possibility of investigating the functional role of the stomatal morphology of cycads. Moreover, we show an unexpected degree of variation between subclades in the family, potentially connected to differences in whole leaflet development, and validate hypothesis of convergent evolution of the stomatal morphology in the Stangeriaceae, while strengthening the relationship between *Stangeria* and the *Ceratozamia*–*Zamia*–*Microcyas* clade.

### Mature stomatal structure

**Fig. 9.**
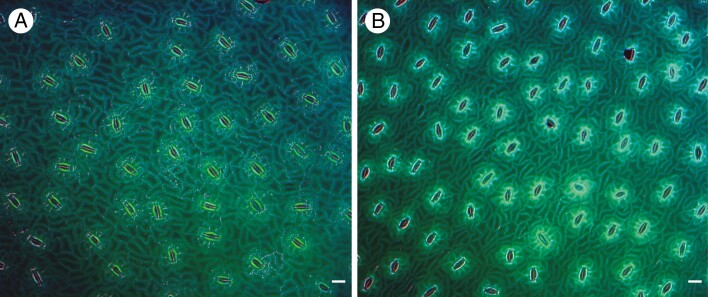
(A) *Cycas circinalis* and (B) *Cycas thouarsii*; mature isolated cuticles stained with Auramine O. Scale bar = 50 µm.

Our comparative observations confirm those of earlier researchers (e.g. Florin, 1931, 1933) in demonstrating the presence of a more or less distinct ring of subsidiary cells and polar cells in most extant Zamiaceae (Fig. 2). A similar arrangement also occurs in the sister genus *Cycas* (Fig. 9), in which both mature stomata and stomatal development have previously been described (Pant and Nautiyal, 1963; Pant and Mehra, 1964; Griffith *et al.*, 2014). The stomatal apparatus of *Bowenia* differs in lacking encircling cells in a mature stomatal complex due to the lack of divisions of the lateral neighbour cells (Coiro and Pott, 2017). Based on mature leaflet anatomy, Coiro *et al.* (2020) suggested that this heterochronic difference could be due to relatively rapid elongation and differentiation of the pavement cells during leaflet development in *Bowenia*. Our study shows that axial elongation is synchronous with GMC division in *Bowenia*, and that the neighbour cells undergo divisions in all species examined except *B. spectabilis.*

In most cycads, mature stomata are predominantly axially orientated with their apertures more or less parallel to the long axis of the leaflet (with some exceptions; see below and Table 3). This pattern resembles the condition in most other land plants (e.g. conifers) but contrasts with fossil Bennettitales, in which the apertures are transversely orientated (Rudall and Bateman, 2019). GMCs are more or less square in all species examined and no asymmetric divisions were observed, indicating the absence of amplifying divisions that would lead to random stomatal orientation. A consistent axial orientation indicates linear epidermal expansion and an absence of amplifying divisions.

**Table 3.** Summary of stomatal patterning and development in the ten extant cycad genera

Species	Mature leaf epidermis (this paper; Florin 1931, 1933; Greguss 1957, 1968)	Mature stomata (this paper; Florin 1931, 1933; Greguss 1957, 1968)	Stomatal differentiation and pre-patterning
*Bowenia*	Pavement cells axially aligned but relatively short and sometimes irregular	Stomata not sunken or only slightly sunken; axially orientated	Subsidiary cells perigenous; stomatal development synchronous; linear pre-patterning (this paper)
*Ceratozamia*	Pavement cells narrow and axially elongated	Stomata sunken; axially arranged and orientated; sunken	Protodermal cells isodiametric (this paper); ‘quartet’ pre-patterning (this paper)
*Cycas*	Pavement cells isodiametric with irregular alignment	Stomata sunken; mostly similarly orientated but some random	Subsidiary cells perigenous; some stomata formed at later stages (Pant and Mehra 1964); ‘quartet’ pre-patterning (Coiro and Pott, 2017)
*Dioon*	Pavement cells axially aligned but relatively short and sometimes irregular	Stomata sunken; axially orientated	Subsidiary cells perigenous; stomatal development synchronous (this paper; Florin 1931)
*Encephalartos*	Pavement cells irregular	Stomata sunken; axially orientated	Unknown
*Lepidozamia*	Pavement cells axially aligned	Stomata sunken; axially orientated	Unknown
*Macrozamia*	Pavement cells axially aligned or isodiametric; relatively short	Stomata slightly sunken; axially orientated	Subsidiary cells perigenous; stomatal development synchronous (this paper)
*Microcycas*	Pavement cells short, irregular	Stomata sunken; axially orientated	Unknown
*Stangeria*	Pavement cells with sinuous anticlinal walls	Mostly axially orientated, but some random; not sunken	Subsidiary cells perigenous; some stomata formed at later stages (this paper)
*Zamia*	Pavement cells axially aligned but relatively short and irregular	Axially oriented; sunken	Subsidiary cells perigenous; stomatal development synchronous; ‘quartet’ pre-patterning (this paper)

Although highly speculative, the consistent presence in cycad stomata of subsidiary cells, often with persistent cytoplasm and nucleus (unlike the almost completely sclerified pavement cells), could indicate a physiological role. A physiological connection between the guard cells and subsidiary cells is well known in grasses, where the lateral subsidiary cells are involved in the mechanisms of stomatal opening via exchange of osmolytes with the guard cells (Franks and Farquhar, 2007; Raissig *et al.*, 2017; Gray *et al.*, 2020). Some authors have reported a relatively rapid mechanism of closure and opening and high water efficiency in the Cycadales (Haworth *et al.*, 2011; Álvarez-Yépiz *et al.*, 2017). On the other hand, encircling cells and polar cells are almost completely sclerified at maturity in all genera except *Stangeria*, in which they contain oxalate crystals.

Mature stomata are sunken in stomatal pits in all species examined except *B. serrulata* and *S. eriopus*, in which the guard cells lie at the same level as surrounding epidermal cells or are only slightly sunken. As we have demonstrated, and Florin (1931) also demonstrated in *D. edule* (Fig. 2), the stomatal pit results from anticlinal enlargement of the subsidiary cells and the polar cells, often followed by division of the subsidiary cells. In *S. eriopus*, the initial division is periclinal, while it is oblique in the other species. In *D. edule*, the precursor of the encircling cells divides further, resulting in a three-layered encircling chamber in all members of this genus (Barone Lumaga *et al.*, 2015).

The ‘flush’ guard cells of *Stangeria* and *Bowenia* result from differing ontogenetic paths (Table 3), strengthening the hypothesis of parallel evolution for this supposed synapomorphy (Coiro and Pott, 2017; Coiro *et al.*, 2020). This convergent morphology could be linked to leaflet economics in these two genera. Both genera share a similar habit, with subterranean stems, few large leaves and growth mainly in shaded areas, although both *Bowenia* and *Stangeria* can also grow in sunny habitats. The sunken guard cells of most cycads have traditionally been associated with adaptation to aridity. In some genera, such as *Dioon* (Barone Lumaga *et al.*, 2015; Vovides *et al.*, 2018), the depth of the stomatal pits is associated with plants living in more xeric environments. However, it is unclear whether the depth of the pits is an adaptation to avoid water loss, since there is little direct evidence either from models (Roth-Nebelsick *et al.*, 2009) or from comparative analyses of other groups (Jordan *et al.*, 2008). Deep stomatal pits could be a way of reducing the already elevated mesophyll resistance provided by the thick cells walls of gymnosperms (Veromann-Jürgenson *et al.*, 2017; Carriquí *et al.*, 2020). Thicker leaves, which are potentially adaptive in arid climates, would necessitate deeper pits, in a similar fashion to the evolution of crypts in sclerophyllous taxa (Hassiotou *et al.*, 2009). *Bowenia* and *Stangeria* have among the thinnest leaves in the Zamiaceae, and thus lack stomatal pits.

### Stomatal development

Our comparative observations show that in Zamiaceae the stomatal subsidiary cells are derived from protodermal cells adjacent to the GMC, rather than from the sister cell to the GMC. In a few cases where epidermal cell elongation precedes GMC formation, we did observe that one of the polar cells is mesogenous, for example in *M. communis* (Fig. 6A), where the GMC is apparently the sister to the adjacent cell. Indeed, as Rudall and Bateman (2019) noted, in narrow linear leaves with axially elongated cell files, the GMC is invariably sister to one of the polar neighbour cells, as also observed in conifers (*Pinus*: Johnson and Riding, 1981) and monocots (Rudall *et al.*, 2017). However, in most cycad species the protodermal cells are isodiametric and remain relatively short.

This perigenous pattern of development agrees with the haplocheilic definition of Florin, and matches studies of early stomatal development in *Cycas*, *Ceratozamia* and *Dioon* (Florin, 1931, 1933; Pant and Mehra, 1964; Barone Lumaga *et al.*, 1999). A similar pattern of development was also reported in several other extant gymnosperms, including conifers (Florin, 1931; Johnson and Riding, 1981) and *Ephedra* of Gnetales (Rudall and Rice, 2019), but not in other Gnetales (*Gnetum* and *Welwitschia*: Takeda, 1913*a*, *b*; Rudall and Rice, 2019), in which the subsidiary cells are clearly derived by division of the meristemoids. In *Ginkgo*, which has fan-shaped leaves with relatively chaotic epidermal patterning, both perigenous and mesogenous neighbour cells are observed (Rudall *et al.*, 2012).

We also observed some differences among extant Zamiaceae in epidermal patterning and pre-patterning. The most common condition within the family is axial patterning with consistent orientation of stomata and consistent timing of the development of adjacent stomata (Table 3). For example, in *D. edule* and *M. communis*, the epidermal precursor cells are already arranged in clear axial files. In *B. spectabilis* (Fig. 2), axial cell files are discernible but less regular during early pre-patterning, with occasional transverse divisions; elongation of the pavement cells occurs before or synchronously with GMC division, and thus precedes guard-cell differentiation in this species. GMCs differentiate almost synchronously in the same section of the leaflet in *Dioon*, *Bowenia* and *Macrozamia*.

In contrast, ‘quartet’ pre-patterning, in which groups of four protodermal cells occur in a squared arrangement (Rudall and Knowles, 2013; Rudall and Bateman, 2019), is present in some Zamiaceae (*Stangeria*, *Ceratozamia*, *Zamia*) (Table 3) and also in *Cycas* (Coiro and Pott, 2017). Within Zamiaceae, this correlation provides support for a potential relationship between *Stangeria* and the *Ceratozamia*–*Zamia*–*Microcycas* clade, hypothesized from molecular and anatomical data (Salas-Leiva *et al.*, 2013; Coiro *et al.*, 2020). In the *CSMiZ* clade, including *Ceratozamia hildae* (Fig. 2), *Zamia integrifolia* (Fig. 6) and *Stangeria eriopus* (Fig. 5), axial cells files are absent at early stages.

*Stangeria* is also unusual within Zamiaceae in that new GMCs differentiate during later development, often at different orientation to the early-formed stomata, resulting in a close proximity of stomatal complexes at different stages of development (Fig. 7E, F). The presence of both ‘quartet’ pre-patterning and successive development is partly correlated with leaf development. In *Stangeria*, growth of the leaf lamina starts from a continuous marginal meristem that develops after elongation of the leaflet midrib. In *Zamia* and *Ceratozamia*, the leaflet primordia undergo not only longitudinal expansion, but also lateral expansion that results in variation in leaf width between species (Medina-Villarreal *et al.*, 2019).

*Stangeria* shares with *Cycas* non-synchronous stomatal differentiation and inconsistent stomatal orientation (Table 3). Previous observations on *Cycas* leaflet development as well as the mature shape of the epidermal cells show a ‘quartet’ pre-patterning (Coiro and Pott, 2017), indicating that this pattern could be either ancestral in cycads or occurred independently in *Cycas* and the *CSMiZ* clade. However, the absence of a clear outgroup for cycads makes resolution of this issue currently unfeasible. Preliminary inferences suggest that fossil cycads also show both squared and linear pre-patterning, but difficulties in placement of these fossils in the cycad phylogeny (Erdei *et al.*, 2019) make determining character polarities highly problematic. The long evolutionary time span between the origin of Cycadales and crown-group Zamiaceae and Cycadaceae limits our ability to infer character history using outgroup comparison.

## CONCLUSIONS

Although the perigene origin of subsidiary cells in cycads is confirmed by our results, the division of the lateral cells into subsidiary and encircling cells suggests that the traditional separation between ‘haplocheilic’ and ‘syndetocheilic’ stomata fails to fully capture the variability in stomatal development and morphology observed between the extant gymnosperm groups, especially following translation in different languages. This potential difficulty in making accurate comparisons, together with recent new observations on stomatal development in other extant and extinct groups (Rudall *et al.*, 2013; Rudall and Bateman, 2019; Rudall and Rice, 2019), suggests that a revision of the stomatal characters in the morphological matrices of the seed plants (Doyle 2006; Hilton and Bateman 2006) might be necessary to improve the resolution of the relationships between these groups.

The similarity between early development of the epidermis in cycads and other gymnosperms suggests that the lack of response of cycad stomatal density to CO_2_ (Haworth *et al.*, 2011) might not be linked to developmental constraints. Further physiological studies are needed, including electrophysiological investigations, stomatal mechanics, and response to desiccation or abscisic acid, to test whether the responses of cycad stomata differ radically from those of the more efficient angiosperm stomata.
